# Animal Models of Vestibular Evoked Myogenic Potentials: The Past, Present, and Future

**DOI:** 10.3389/fneur.2018.00489

**Published:** 2018-06-25

**Authors:** Brian D. Corneil, Aaron J. Camp

**Affiliations:** ^1^Department of Physiology and Pharmacology, University of Western Ontario, London, ON, Canada; ^2^Department of Psychology, University of Western Ontario, London, ON, Canada; ^3^Robarts Research Institute, University of Western Ontario, London, ON, Canada; ^4^Discipline of Biomedical Science, Sydney Medical School, University of Sydney, Sydney, NSW, Australia

**Keywords:** vestibular physiology, vestibular evoked myogenic potentials, animal models, guinea pig, cat, monkey

## Abstract

Vestibular-evoked myogenic potentials (VEMPs) provide a simple and cost-effective means to assess the patency of vestibular reflexes. VEMP testing constitutes a core screening method in a clinical battery that probes vestibular function. The confidence one has in interpreting the results arising from VEMP testing is linked to a fundamental understanding of the underlying functional anatomy and physiology. In this review, we will summarize the key role that studies across a range of animal models have fulfilled in contributing to this understanding, covering key findings regarding the mechanisms of excitation in the sensory periphery, the processing of sensory information in central networks, and the distribution of reflexive output to the motor periphery. Although VEMPs are often touted for their simplicity, work in animals models have emphasized how vestibular reflexes operate within a broader behavioral and functional context, and as such vestibular reflexes are influenced by multisensory integration, governed by task demands, and follow principles of muscle recruitment. We will conclude with considerations of future questions, and the ways in which studies in current and emerging animal models can contribute to further use and refinement of this test for both basic and clinical research purposes.

## Introduction

The past 25 years have seen remarkable advances in the clinical assessment of the vestibular system, driven by improved understanding of the clinical importance of detecting end-organ deficits. Previously, assessment suffered from the inability to specifically test otolith function, hence clinical and ancillary vestibular end-organ testing was disproportionately focused on the horizontal canal through the use of caloric and rotary stimuli. Additionally, horizontal canal assessment could be unpleasantly vertiginous in the case of a caloric stimulus, or require fairly expensive and/or axis-limited setups in the case of rotary chair tests. While these tests still have their place in the clinic, a new generation of relatively simple and inexpensive tests now rapidly screen overall vestibular function without inducing unpleasant vestibular percepts. Assessments of otolith function through the use of vestibular-evoked myogenic potentials (VEMPs) are a core component of this battery of tests. In these tests, transient pulses of air-conducted sound (ACS) or bone-conducted vibrations (BCV) vibrations are delivered repeatedly, and stimulus-triggered averages are generated of the response to this stimulus on neck muscles in the case of the *cervical VEMP (cVEMP*) or extraocular muscles in the case of the *ocular VEMP* (oVEMP). The timeframe and recruitment profile of cVEMPs and oVEMPs are well-established and repeatable, evolving within < 25 ms if the vestibular system is healthy. Deviance from the expected profile, either in term of the response being absent, delayed, or evoked by abnormal stimulus parameters may be indicative of an underlying pathology that requires further investigation. For example, cVEMPs in healthy individuals are evoked by sound intensities in the order of 120–125 dB SPL, but can be evoked at much lower sound intensities consequent to superior canal dehiscence (1), which can produce Tullio phenomenon of sound-induced vertigo or eye movement nystagmus (2). Likewise, the presence or absence of VEMP responses has been well correlated with motor function and development in children with profound sensorineural hearing loss (3, 4).

VEMPs are cost-effective and easy to administer in the clinic, although users should be aware of technical pitfalls that can falsely indicate vestibular pathology if the test is not performed properly (e.g., ensuring adequate baseline muscle contraction, delivering a sufficient but not damaging level of sound intensity). Such concerns are all the more pertinent when assessing an elderly cohort, given that VEMP response rates can drop to 60% in healthy subjects older than 60 years old (5, 6). VEMPs also have a role in basic and clinical research, as VEMPs arise through a brainstem reflex that is itself both context-dependent and highly multimodal; as an example, VEMPs are modulated by increased fear and anxiety, attesting to inputs from centers processing emotional and affective information into the vestibular nucleus (7, 8). Recent reviews (9, 10) have summarized the clinical significance of these tests in parallel with other tests of vestibular function, and outlined best practices for conduct of these tests in the clinic and the laboratory.

Early observations of evoked potentials recorded from the human scalp following loud clicks suggested that they were of cortical origin, possibly arising from activation of the deep auditory cortex (11). Subsequent work by Bickford et al. (12, 13) established the myogenic nature of this earliest phase of the response (within 25 ms of the sound stimulus) across a variety of muscles in the dorsal neck, and showed the dependency of such responses on the degree of tonic muscle recruitment. Anecdotal work by this group in patient populations suggested that the response persisted in patients with hearing pathologies but disappeared in patients with vestibular pathologies, implicating activation of the vestibular apparatus.

Modern assessments of the cVEMP are largely based on the work of Colebatch et al. (14) who, unlike Bickford and colleagues, recorded bilaterally from the sternocleidomastoid (SCM) muscle on the ventral aspects of the neck. SCM offered a more specific recording target, and bilateral recordings permitted the assessment of the laterality of the cVEMP response on this muscle. In a cohort of subjects ranging in age from 29 to 63, unilateral ACS reliably elicited a cVEMP on the ipsilateral SCM relative to the side of the sound; although responses often (but not always) evolved on the contralateral SCM, such responses tended to be much smaller in magnitude. These authors characterized the timing of the cVEMP on the ipsilateral SCM, describing positive then negative peaks appearing at 13 and 23 ms, respectively (the p13 and n23 peaks), and showed that cVEMP magnitude (i.e., the difference between the positive and negative components) rose as a linear function of background activity. Finally, work in selected patients confirmed that the cVEMP persisted in individuals with sensorineural hearing loss but was abolished in individuals with surgical section of the vestibular nerve for treatment of intractable vertigo. Since this study, the vast majority of cVEMP studies have recorded from SCM, concentrating almost exclusively on the response on the SCM ipsilateral to the side of sound presentation.

In parallel with these observations in humans, work in animal models refined the understanding of basic vestibular function, and addressed the heterogeneity of vestibular afferents innervating the vestibular end organs [for review see (15)]. Seminal work conducted by Young et al. (16) in the squirrel monkey established the comparative sensitivity of particular vestibular afferents originating from the otolith rather than the canals to sinusoidal patterns of both ACS and BCV. Subsequent work in the vestibular systems of guinea pigs studied the responses of primary vestibular afferents to ACS (17–19) or BCV (20). The latter work contributed to the understanding and development of the oVEMP in humans (21, 22). Together, such research in human and animal models illustrated that the vestibular end-organs can be activated by sound or vibrational stimuli, laying the foundation for a component of modern vestibular assessments.

In this review, we will summarize key results in animal models that have contributed to the understanding of the functional anatomy and physiology underlying VEMPs at a resolution that could not have been achieved in humans. We will focus primarily on cVEMPs provoked by ACS, although we will occasionally stress key responses related to oVEMPS arising from BCV. Our rationale for doing so parallels the earlier adoption of cVEMP testing in humans. Further, the comparative anatomy across animals and humans is far more complicated for cephalomotor vs. oculomotor control. Our review will focus on three main topics and animal models: (i) the means by which sound waves excite otolith receptors, which will primarily focus on results in guinea pigs, (ii) the integration of vestibular information within the vestibular nucleus, which will primarily highlight results from cats, and (iii) the distribution of motor commands to neck muscles, which will primarily consider results in monkeys. In doing so, we aim to illustrate the ways in which work in animal models have contributed indispensible information to modern vestibular assessments, We will also discuss how work in animal models illustrate the complexity in the functional anatomy and physiology of this seemingly simple evoked response. This is particularly relevant within the clinical realm, as it moves beyond diagnostic vestibular end-organ testing to consider the functional implications of such deficits and how they may be rehabilitated in humans.

## The action at the sensory apparatus

VEMPs arise from a form of sensory cross-talk, in that the sensory receptor is activated by energies arising from a stimulus other than that to which it is most sensitive. In the case of ACS, the earlier work in human subjects referenced above provided hints that activation may be arising at the otolith organs, and more specifically at the saccule, but a more precise understanding of the underlying mechanism was not possible in this preparation.

As mentioned above, fundamental work on the responses of vestibular afferents to sound and bone vibration was conducted in the squirrel monkey (16). In this study, the authors applied long-duration (3 s or more) sinusoids of ACS or BCV across a range of stimulus intensities and frequencies while recording from vestibular nerve fibers innervating both the canals and otoliths. Although responses could be driven from all end-organs, afferents from otolith organs were on average more sensitive to ACSs than those from canals, being activated at lower stimulus intensities. Importantly, the phase in which a given saccular afferents responded to the sound stimulus varied systematically with the functional polarization of the afferent (defined as the responsiveness to upward or downward static tilts). The relevance of this observation is that it implicates activation of hair cells systematically organized on the macula via a fluid wave mechanism, rather than direct activation of the vestibular afferent.

In terms of responses to BCV, Young and colleagues observed that afferents with irregular discharge rates (termed IA afferents) tended to be more sensitive to BCV than afferents with regular discharge rates (RA afferents). However, and somewhat surprisingly in light of later work, canal afferents were reported to be more responsive than otolith afferents in Young et al. (16). It is important to recognize that responsiveness in the context of this study was primarily defined as the appearance of phase-locking of the afferent response, rather than a change in firing rate *per se*. Further, such observations held for lower vibration frequencies than those used in subsequent studies, and in the clinic.

**Figure 1:**
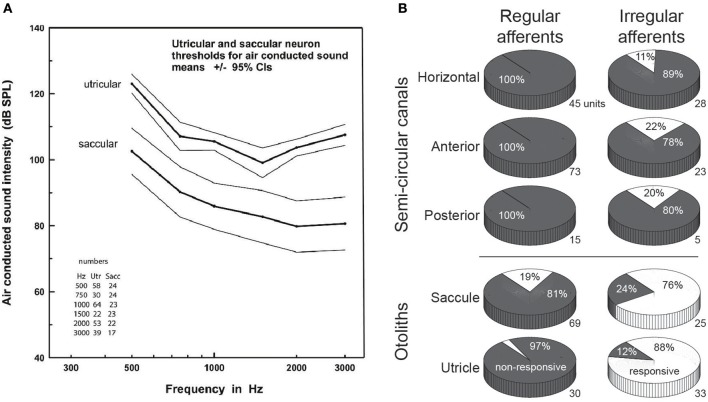
**(A)** Response thresholds for utricular vs. saccular afferents to various frequencies of air-conducted sound. Note the lower thresholds for saccular vs. utricular afferents. Reproduced from Curthoys et al. (20), with permission from Elsevier. **(B)** Comparative response proportions for regular and irregular afferents from the various vestibular end-organs to bone-conducted vibration in the guinea pig, expressed as a percentage of responsive vs. non-responsive afferents (the number to the bottom-right of each pie chart shows the sample size. Note the greater responsivity for otolith vs. canal afferents, and for irregular vs. regular afferents. Data taken from a table presented in Curthoys et al. (20), with permission from Springer Nature.

The central observation that loud auditory stimuli, whether delivered as transient clicks or prolonged tones, can preferentially (although not selectively) excite otolith afferents has been observed in multiple mammalian species, including the mouse (23), rat (24), guinea pig (17, 19), cat (25), and chinchilla (26). Work in the guinea pig model, conducted in large part by Curthoys and colleagues, provides the most comprehensive description of otolith activation by ACS [see 27 for a comprehensive recent review of this and related work]. Over numerous physiological studies that have recorded the activity of single vestibular afferents, it is now firmly established that ACS at clinically-relevant intensity levels almost exclusively activates afferents stemming from otolith organs, with afferents from the saccule being on average more sensitive than those from the utricle (Figure 1A). Moreover, as originally observed by Young et al. in the squirrel monkey (16), it is the IA afferents that are most sensitive to ACS. Similarly, BCV preferentially activates IA rather than RA afferents stemming from otolith organs (20, 28) (Figure 1B).

Work in animal models has proven essential in addressing a number of key questions about VEMPs. First, what is it about the IA afferents that render them preferentially sensitive to ACS or BCV compared to RA afferents, and why are saccular rather than utricular IA afferents on average more sensitive to ACS? The answers appear to be found in an elegant combination of anatomy, physiology, and fluid dynamics [see (27) for review]. Fundamentally, both ACS and BCV set up a fluid wave within the endolymph of the inner ear. However, the fluid wave consequent to ACS begins at the oval window, and hence has a more direct path to the saccule rather than utricle, which may be why ACS preferentially activates saccular rather than utricular afferents (29). Regardless of whether it stems from ACS or BCV, a fluid wave transmitted to the otoliths appears to deflect the hairs of type I hair cells that are preferentially distributed along the striola of the otolithic macaula and are both shorter than the hairs of type II hair cells innervated by RA afferents and more loosely coupled to (or even free-floating from) the overlying otolithic membrane (30, 31). Consequently, fluid waves arising from either ACS or BCV deflects striolar Type I hair cells to initiate signaling preferentially along IA rather than RA afferents, particularly when delivered at lower energies (29).

A second question pertains to the greater sensitivity of IA afferents stemming from the otoliths rather than canals. Work in a variety of animal models stresses the importance of an intact bony labyrinth in conferring such preferential sensitivity; normally the bony labyrinth prevents the fluid wave from activating canal afferents except at very high energy. Artificial induction of a dehiscence, or opening of the bony labyrinth alters the sensitivity of canal afferents to ACS and BCV (23, 26, 29). Effectively, the dehiscence provides an alternative path for flow of the fluid wave, to which the sensitive IA afferents from the canals can now respond. Such work provides an animal model for Tullio's phenomenon, wherein nystagmus can be provoked in phase with louds sounds due to a third window within the labyrinth.

An understanding of the mechanism activation with ACS or BCV stands as a useful point of contrast to another common means to activate vestibular afferents and induce VEMPs, via galvanic vestibular stimulation. In humans, electrical current is applied directly at the mastoid, activating the nearby vestibular nerve. Work in the monkey (32) and guinea pig (33) has shown that primary afferents from both the otoliths and semi-circular canals that are activated by galvanic vestibular stimulation, with IA afferent being activated at the lowest currents. Accordingly, there are key differences both at the site of activation with galvanic vestibular stimulation vs. ACS or BCV (with the former method activating the primary afferents and hair cells, and the latter methods influencing only the hair cells), as well as in the comparative sensitivity of the otoliths vs. canals to these forms of stimulation. The considerations impact the use of these tests in both the lab and clinic, and the interpretation of any results. For example, the comparative site of activation can help differentiate pathologies at the hair cells from those influencing the vestibular nerve (34).

## Central integration within the vestibular nuclei

**Figure 2:**
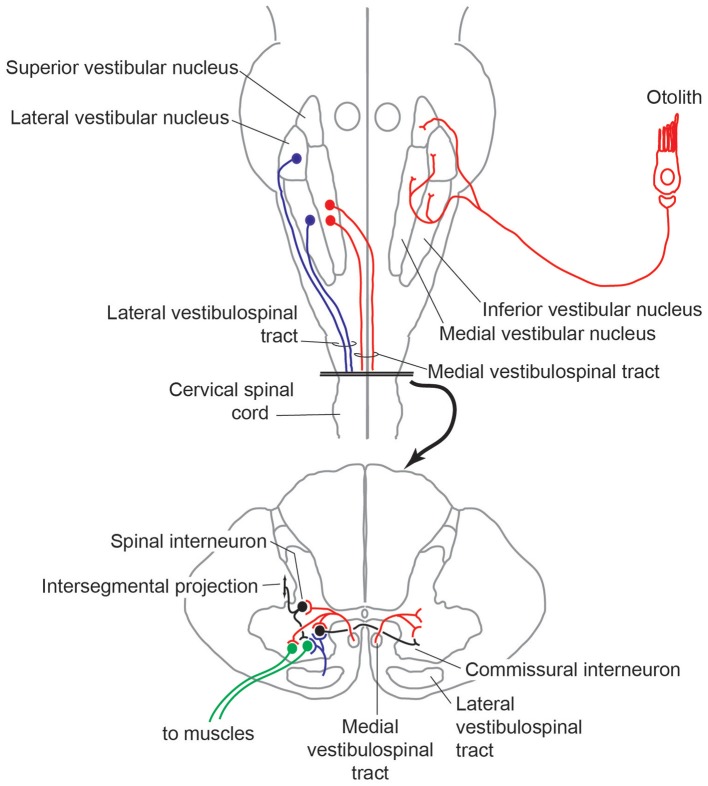
Trajectory of medial and lateral vestibulospinal tracts, and interface within intrinsic spinal circuits.

The basic circuit underlying both the cVEMP and oVEMP is the same three-neuron arc that underlies the vestibulo-cervical (VCR) and vestibulo-ocular (VOR) reflex, respectively. Although the exact pattern of projections of IA afferents responding to ACS or BCV to the vestibular nucleus has not been determined, otolith afferents in general ramify widely within the vestibular nuclei (15, 35–37), with projections (amongst many other targets) to areas containing second order neurons contributing to vestibular reflexes. Descending vestibular information is relayed through the spinal cord via either the ipsilateral or contralateral lateral vestibulospinal tract (LVST) that takes origin from the lateral and inferior vestibular nuclei), or the medial vestibulospinal tract (MVST) that takes origin mainly from the medial vestibular nucleus. The MVST mainly terminates in cervical segments, and is essential in mediating the cVEMP and VCR, whereas the LVST extends down to spinal segments, and hence mediates VEMP responses on muscle groups below the neck and in vestibulospinal reflexes more generally (Figure 2).

Work conducted in many animal models has been essential in delineating these reflexive circuits, and highlighted the ways in which vestibular information is integrated with other sensory sources and with other descending motor control pathways in order to contribute to the body's overall sense of position and equilibrium and coordinate a whole-body response. Information from vestibular receptors can also access neck muscle motoneurons via reticulospinal and interstitospinal pathways that receive inputs from the vestibular nuclei and lie in parallel to the MVST and LVST (38). Further, all descending motor control pathways ramify not only into the ventral horn containing the neck muscle motoneurons, but also into the intermediate layers of the spinal cord containing segmental, intersegmental, or commissural spinal interneurons (39). Within the broader context of overall body function, vestibular reflexes must be temporarily gated or attenuated during volitional movements (40), otherwise they would be counter-productive, and there is evidence from the guinea pig that the VOR can be driven by an anticipatory response that is in synchrony with an intentional head movement (41). Readers interested in these broader topics are pointed to a number of recent reviews that describe contextual processing of vestibular information in awake, behaving preparations (42, 43).

Work by Uchino et al. [reviewed in (44)] examined the comparative connectivity of the otoliths to the motor periphery. This work was performed in an anesthetized cat preparation, and combined selective stimulation of individual vestibular nerve branches with functional identification of neurons within the vestibular nucleus based on the presence of connections within the upper brainstem and spinal cord, and intracellular recordings from extraocular or selected neck muscle motoneurons. This preparation, first reported in Sasaki et al. (45) enabled identification of the patterns of di- or tri-synaptic excitation or inhibition following selective stimulation of individual nerve branches, and proved to be particularly useful in distinguishing the differential patterns of projections from the utricle vs. saccule to both the eye and neck. Selective utricular stimulation evoked a pattern of disynaptic excitation on ipsilateral neck muscle motoneurons and trisynaptic inhibition on contralateral neck muscle motoneurons, regardless of whether the muscles under consideration served as presumed extensors or flexors (46, 47). Selective sectioning of descending pathways suggested that these responses were mediated by the ipsilateral LVST, with contralateral inhibition arising from commissural neurons in the upper cervical spinal cord. In contrast to the lateralized patterns of innervation stemming from the utricle, saccular projections evoked patterns of di- and tri-synaptic bilateral excitation on extensor neck muscles and bilateral inhibition on flexor neck muscles (48), with both the ipsilateral LVST and MVST mediating different aspects of these responses. Uchino et al. speculated that the lateralized vs. symmetrical recruitment synergies recruited by utriculo-collic vs. sacculo-collic reflexes, respectively, related to their comparative reference frames in which they operate.

**Figure 3:**
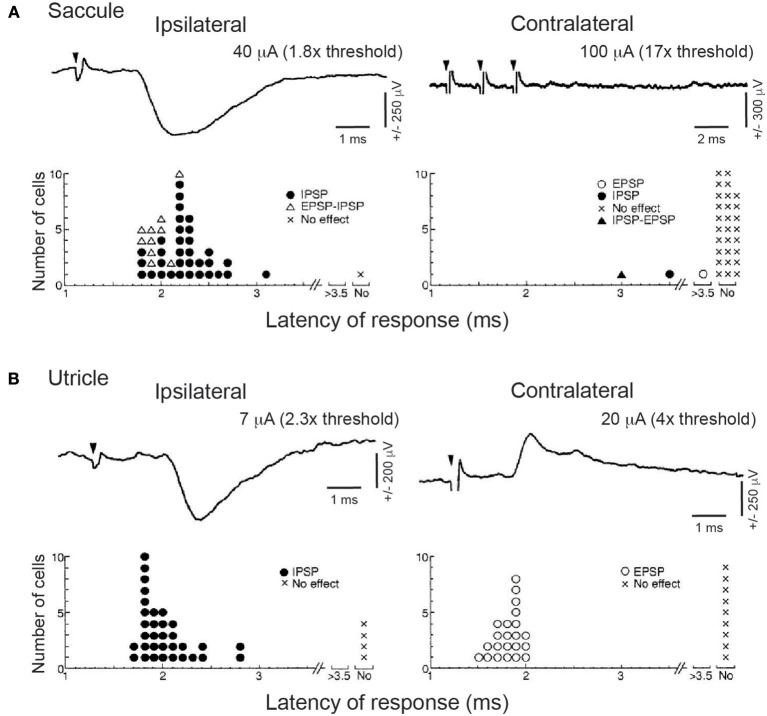
Effects of saccular **(A)** or utricular **(B)** stimulation on ipsilateral or contralateral SCM motoneurons recorded intracellularly. Upper rows of **(A)** and **(B)** show typical waveform or IPSPs (upper left) or EPSPs (upper right of **B**) evoked by selective nerve stimulation; note the absence of any effect following even three pulses of saccular stimulation (downward filled triangles show time of stimulation pulses). Stimulation currents relative to nerve threshold. Lower rows of **(A)** and **(B)** show histograms summarizing response type and response latency across all recordings, emphasizing the selective ipsilateral effect of saccular stimulation and the bilateral effect of utricular stimulation. Reproduced from Kushiro et al. (51), with permission from Springer Nature.

As will be described in more detail below, the functional anatomy and physiology of the neck is extremely complicated. In the cat, more than 20 pairs muscles can potentially contribute to head motion, with a given muscle contributing to many different motions (49, 50). This leads to a degree of redundancy, in that a given head motion could be brought about by an almost unlimited combination of patterns of neck muscle recruitment. Uchino and colleagues identified the specific neck flexor they were recording [e.g., longus capitis (LC) in (47) and (48)], or that they were recording form nerve roots at the upper cervical levels that mainly, although not exclusively, innervate the neck extensors biventer cervicis (BC) and complexus (COM) (46, 48). The importance of knowing the specific neck muscle being recorded became apparent when Uchino and colleagues examined the connections from the otolith organs to SCM (51), a large and powerful ventral neck muscle that contributes to both flexion and contralateral head rotation in the cat (49, 50). Saccular projections to SCM appear to be the exception to the rule, in that saccular nerve stimulation induced disynaptic inhibition of ipsilateral SCM but did not induce a response on contralateral SCM (Figure 3A). In contrast, selective utricular nerve stimulation induced a lateralized pattern of disynaptic inhibition of ipsilateral SCM and disynaptic excitation of contralateral SCM (Figure 3B). Transection experiments suggested that these responses were mediated primarily by the MVST. Related work described a strong effect of selective utricular stimulation on the inferior oblique extraocular muscle, but a comparatively weak or absent effect on this muscle following selective saccular stimulation (52, 53).

These differences in the comparative neck and extraocular muscle responses following selective saccular or utricular nerve stimulation are often cited as key findings for the neural basis of VEMP testing in humans. However, it is important to recognize that this work, while essential, investigated selected neck muscles in an anesthetized cat preparation via stimulation of almost the entire saccular or utricular nerve that presumably activated both IA and RA afferents. Hair cells on the utricle or saccule are variably oriented to transduce linear acceleration or tilt in all possible directions in the horizontal or vertical plane, hence opposing reflexive responses at the neck should be induced by motion in opposing directions; to put it another way, presumably differential patterns of responses should be evoked from hair cells innervating different parts of the otolithic macula. Further, connections from the saccule within the vestibular nucleus exhibit cross-striolar inhibition (54), so that excitatory inputs originating from one portion of the saccule (preferentially representing one direction of motion) inhibit second-order vestibular nucleus neurons receiving afferent input from that portion of the saccule representing the opposite direction of motion.

**Figure 4:**
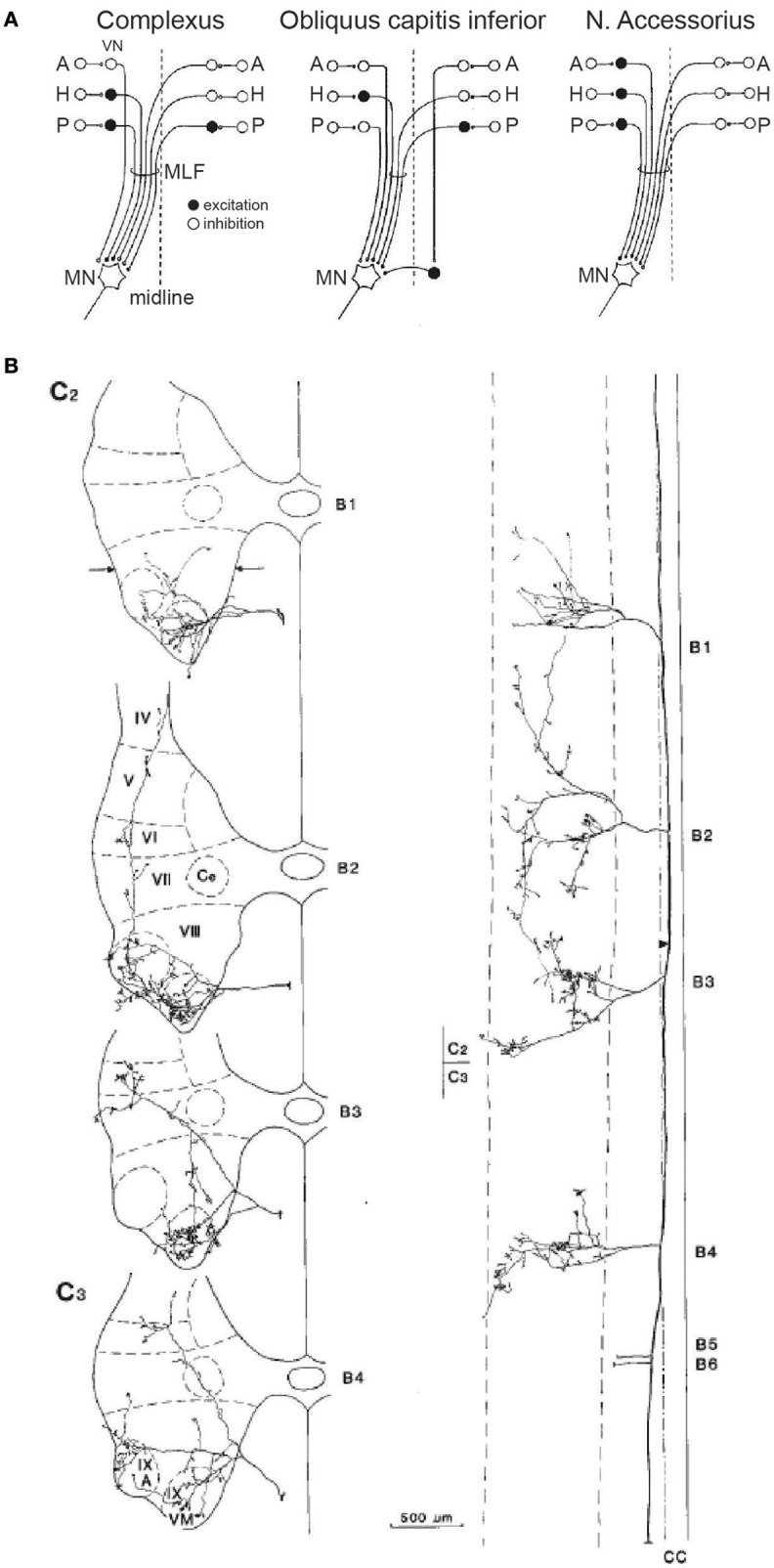
**(A)** Convergent input patterns and pathways from the six semicircular canals onto motoneurons (MN) supplying three different neck muscles. Open and closed circles represent excitatory and inhibitory neurons, respectively. VN, vestibular nucleus; A, H, P, anterior, horizontal, and posterior canals; MLF, medial longitudinal fasiculus; N, nucleus. **(B)** Distribution of an uncrossed neuron in the MVST, showing projection patterns in the transverse (left) and horizontal (right) plane at the C2 and C3 levels. Note distribution of terminals throughout Rexed's laminae IV–IX, and the ramifications across multiple levels of the cervical spinal cord. VM, ventromedial nucleus; A, accessory nucleus. Reproduced from Sugiuchi et al. (61), with permission from Elsevier.

Many second-order neurons contributing to the VCR receive converging projections from both canals and otolith afferents (55–58); second-order vestibular neurons also receive converging inputs from both the utricle and saccule (59). Such convergence is thought to help resolve the tilt-translation ambiguity present in the responses of primary otolithic afferents (60), and coordinate the responses to real-world perturbations that are not conveniently constrained to the purely horizontal or vertical dimension. Shinoda and colleagues have conducted a series of painstaking anatomical and physiological experiments in the cat to determine the pathways and influence of input from the six semicircular canals to 16 different neck muscles [reviewed in (61)]. In physiological studies, they found that all neck muscle motoneurons received input from each of the six canals, and that all motoneurons within a given neck muscle received one of four patterns of input from the canals, three of which are shown in Figure 4A (62–64). In other anatomical studies, Shinoda and colleagues injected identified LVST and MVST neurons with an anatomical tracer, enabling them to trace axon collaterals from identified neurons to multiple spinal segments innervating separate muscles, and describe the elaboration of such collaterals throughout the ventral horn containing motoneurons and into intermediate layers containing spinal interneurons (65, 66). Figure 4B shows the ramifications of a single uncrossed identified MVST neuron that receives canal input into multiple levels of the spinal cord, elaborating into not only the accessory nucleus that innervates SCM, but also into the ventral horn from which the motoneurons of other muscles, such as BC, COM, and rectus capitis posterior major (RCPmaj), are found. This innervation pattern was found in almost all of the tested uncrossed MVST neurons that received canal input (61).

Shinoda and colleagues found that canal inputs mapped onto a particular muscle in a systematic and repeatable fashion, and also that single LVST and MVST neurons innervated a functionally relevant synergy of multiple neck muscles distributed over multiple spinal segments. Such hardwiring of muscle synergies implements a canal-dependent head movement response, simplifying the neural control of the head by specifying particular recruitment synergies. Other descending motor control systems, such as the tectospinal and reticulospinal systems, appear to tap into the same recruitment synergies (67). Determining exactly how second-order neurons receiving otolith input maps onto multiple neck muscles remains to be determined, and this is a far more complicated question given the representation of multiple directions of motion across the otolithic macula. However, given the otolith-canal convergence noted above, it is reasonable to surmise that otoliths will also be hardwired to directionally-appropriate neck muscle synergies through patterned vestibulo-collic projections.

Although the work by the Uchino and Shinoda groups studied mostly different sets of neck muscles, there is an interesting comparison when it comes to SCM, the traditional target for cVEMP recordings. SCM is innervated by the accessory nucleus, which also innervates the trapezius (TRAP) muscle. While the Shinoda group did not explicitly differentiate between SCM and TRAP, recordings from accessory nucleus motoneurons showed that stimulation of the anterior and posterior canals produced a pattern of disynaptic inhibition and disynaptic excitation from the ipsilateral and contralateral canals, respectively (Figure 4A, rightmost panel). This profile differs from the selective saccular input onto SCM from the ipsilateral but not contralateral saccule shown by Uchino (shown in Figure 3A), even though many of the vestibular nucleus neurons that comprise the MVST receive converging inputs from the saccule and the posterior canals (68), and ramify to multiple spinal levels. It may be the case that SCM and TRAP receive different patterns of innervation from the vestibular nucleus, or that saccular and posterior canal inputs remain segregated within a subset of VN neurons that project to the accessory nucleus. Regardless, and even in light of the apparent selective influence of saccular stimulation on the ipsilateral but not contralateral SCM in the cat found by the Uchino group, any saccular-derived signal descending through the MVST distributed widely both to the motoneurons of multiple neck muscles and into the intermediate layers of the spinal cord as well (69, 70). Many commissural interneurons within the spinal cord also receive vestibular input (71). Such widespread ramifications of descending vestibular signals to multiple targets may be why ACS often provokes cVEMPs on both the ipsilateral and contralateral SCM, and on other muscles throughout the body.

## Distribution of descending commands in the motor periphery

In the final section of this review, we turn to the contribution of animal models to our understanding of the neural control of head motion in humans. Across the animal kingdom, head motion or stabilization fulfills a number of roles, serving not only vestibular reflexes and overall body equilibrium, but also contributing to orienting, feeding, grooming, prehension, emotional expression, and conspecific interactions. Further, neck muscles themselves are also sensory organs, providing information about head-re-body configuration that is needed to transform information obtained from sensors mounted within a mobile head (e.g., eyes, ears, snout, and whiskers) into whole-body actions. The neural solutions for head control, the integration with afferent information with vestibular and other sensory information, and the confluence of descending pathways onto individual neck muscle motoneurons reflect this diversity of function.

Humans have a number of specializations compared to favored animal models that must be acknowledged as they pertain to head control and overall vestibular function, including most obviously bipedal locomotion and foveate vision. While no one animal model incorporates all of these features, the upper cervical column in quadrupedal mammals, including cats, monkeys, rats, mice, and guinea pigs, is orientated vertically due to a marked dorsiflexion at the cervico-thoracic junction; forward flexion at the junction between the skull and upper cervical levels then orients the vestibular apparatus in a common position across mammalian species (72, 73). This organization is thought to help constrain the neuromuscular solutions to move the head in quadrupeds, but such solutions vary across the mammalian kingdom depending on skeletal geometry. For example, the solutions used for example by rabbits to position the head in the sagittal plane are different than those used by humans and monkeys (74); vestibulospinal and other descending pathways likely exploit such constraints to simplify cephalomotor control (72). Fortunately, the overall visual and oculomotor systems in monkeys are very similar to that in humans, and cats also have forward facing eyes and an area centralis specialized for higher visual acuity (75). This is relevant since otolithic reflexes require different interactions between visual and vestibular signals in forward- vs. laterally-eyed animals, depending on viewing distance and vergence (76, 77). Finally, the patterns of locomotion vary across monkeys, the new-world squirrel monkey is an arboreal quadruped, whereas the old-world rhesus macaque is a terrestrial quadrupeds (78). Terrestrial quadrupeds often habitually adopt a sitting posture that frees their forelimbs for manipulation and grooming and resembles aspects of the bipedal posture used in humans (79).

**Figure 5:**
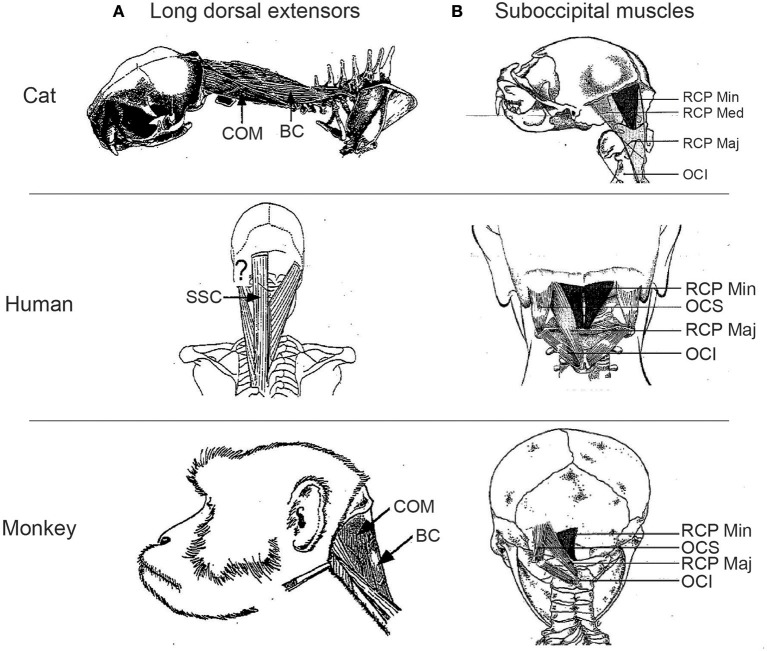
Comparative anatomy of long dorsal extensors **(A)** and suboccipital muscles **(B)** in cats, humans, and monkeys. Question mark in **(A)** shows location of where COM would insert in the human skull, if present. COM, complexus; BC, biventer cervicis; SSC, semispinalis capitis; RCP major, rectus capitis posterior major; RCP min, rectus capitis posterior minor; RCP med, rectus capitis posterior medius; OCI, obliquus capitis inferior; OCS, obliquus capitis superior.

Cats have historically served as the main model for human head control (80), and extensive anatomical and physiological work has been conducted that details the pathways controlling head motion serving vestibular functioning, orienting, and head-on-body proprioception (39, 81, 82). There are marked differences in the comparative anatomy of the head-neck musculature between cats and humans, and the use and attachment of muscles interlinking the skull, scapula, cervical column, and forelimb. For example, while the dorsal neck musculature in both cats and humans consists of four layers of mostly homologously named muscles, muscles like BC and COM do not exist in humans; instead humans have a single semispinalis capitis muscle (SSC) that lacks the more lateral skull attachments typical of COM (question mark in Figure 5A). There are also specializations for quadrupedal vs. bipedal stance and locomotion. For example, rhomboideus capitis that spans from the scapula to the skull in the cat is tonically active during quiet sitting or standing posture (49), but also does not exist in humans (83). Distinctions also appear in the morphometry and associated skeletal geometry of the deepest dorsal suboccipital muscle layer that spans the skull and upper cervical vertebrae, containing the muscles obliquus capitis inferior (OCI), obliquus capitis superior (OCS), and rectus capitis major and minor (RCP maj and RCP min) (Figure 5B). Such differences in musculoskeletal arrangement reflect differences in the pattern of functional recruitment; muscles like RCP maj and splenius capitis (SPL) are often considered as neck extensors in cats, but as ipsilateral head turners in humans. However, caution is always needed in trying to assume recruitment from anatomy alone, as many neck muscles are recruited in non-intuitive ways, particularly when different postures are adopted (49, 84).

There has been a shift toward the rhesus macaque as another model for human head control, driven in part by neurophysiological studies of the oculomotor system due to parallels in how eye-head gaze shifts are generated in humans and monkeys (85, 86), and of the vestibular system (42). The rhesus macaque exhibits a number of features that make it an attractive model, including the use of the hands rather than mouth for many acts of prehension. Further, many of the dorsal neck muscles, including the subocciptial muscle layer (Figure 5B), attain a more human appearance (83, 87), and there is generally good agreement between in the recruitment profile of those muscles that have been studied in both humans and monkeys in similar tasks (85, 88–93). While encouraging, thorough electromyographic (EMG) recruitment studies of many neck muscles have only been conducted while monkeys are in a seated posture (85); the comparative recruitment during quadrupedal stance or locomotion is simply not known, even though such postural changes alter the contribution of the eyes, head, and body to large gaze shifts (94).

Morphometric appearances aside, all neck muscles are composed of hetereogenous muscle fibers and numerous complicated anatomical features, and many neck muscles feature prominent tendinous inscriptions and compartmentalization that challenge the textbook view of a given neck muscle as a single entity. Even the human SCM muscle so commonly targeted for cVEMP recordings is itself composed of four subvolumes, one of which (clediomastoid) runs deep to the other three subvolumes, and hence is not visible from the superficial surface (83). Different muscle compartments may also be recruited independently, as seen in the feline SPL (95), and even the seemingly simple suboccipital muscles in monkeys can feature a surprising degree of variance in the cross-sectional distribution of different fiber types (96). Further, the density of neck muscle spindles is extremely high in the suboccipital neck muscles of humans, monkeys, and cats (96–98), and spindles tend to co-localize in muscle regions with more fatigue-resistant muscle fibers. The significance of this distribution is not known, although the contribution of neck muscle proprioception to overall body equilibrium, and the convergence of these signals onto neurons within the vestibular nucleus, has long been recognized (99).

Many of the deeper muscles of the neck cannot be accurately recorded using surface EMG techniques in humans, as they lie underneath overlying musculature. Other muscles, like SPL, can be recorded with surface techniques in particular locations, but users should be aware of cross-talk from overlying muscles in other locations (100). While deeper neck muscles can be recorded with intramuscular recording techniques in humans (101–103), such studies are typically restricted to specific research questions. Accordingly, much of what we know about neck muscle recruitment across a range of natural behaviors comes from work in animals like the cat (49) and monkey (85), which permit long-term recordings from a multitude of neck muscles during a range of behaviors via chronically-implanted EMG electrodes. Such studies have revealed recruitment synergies similar to that reported by Shinoda and colleagues, although other muscles can be added to this core recruitment synergy depending on posture (84, 85).

These long-term studies in both cats and monkeys have revealed that the recruitment of multiple muscles in this very complex musculoskeletal architecture adhere to a basic principle in motor control to constrain recruitment of powerful muscles comprised of a greater proportion of fatigable muscle fibers only to those tasks requiring greater amounts of muscle force. For example, seated monkeys turn or hold their heads horizontally using a very straightforward strategy, with the suboccipital muscles OCI and RCP maj contributed to all ipsilateral horizontal head turns and off-center postures, to which gradual recruitment of ipsilateral SPL and then contralateral SCM is gradually added only for progressively more eccentric postures or forceful head turns (85). Similar findings hold in cats (49), and for upward and downward head pitches. Thus, although SCM is often considered to be a prototypical contralateral head turning or neck flexion muscle, this muscle often contributes to very little to moderately paced movements or postures. In contrast, robust recruitment of SCM is seen in head-shaking behaviors (49, 85). Such recruitment profiles are entirely consistent with reserving the recruitment of powerful muscles for forceful tasks, given that the feline and primate SCM has a high proportion of fast fibers, and offers a more advantageous moment arm, than the smaller suboccipital muscles that lie closer to the spinal column (87).

**Figure 6:**
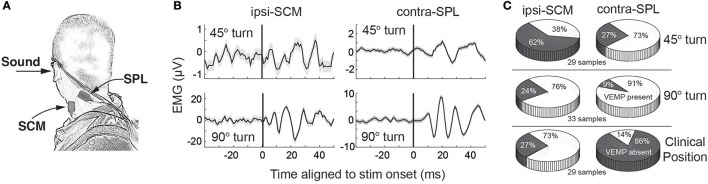
**(A)** Electrode placement to assess cVEMPs from ipsilateral SCM and contralateral SPL. The contralateral SPL electrode is not shown, but is in the mirroring position of that shown here. **(B)** cVEMPs on ipsilateral SCM and contralateral SPL during a modest 45 or more extreme 90 head turn away from the stimulated ear. **(C)** Comparative likelihood of recording a cVEMP on ipsilateral SCM or contralateral SPL for various postures. Same format as Figure 1B. All parts of this figure take from data presented in Camp et al. (107), with permission from Wiley and Sons.

What is the relevance of such observations for cVEMPs in humans? As in cats and monkeys, human SCM also has a higher proportion of fast fibers and a more advantageous moment arm (104–106), leading us to predict that recruitment of this muscle related to vestibular reflexes would also be reserved for tasks requiring larger amounts of muscle force or particularly extreme postures. This line of thinking, as well related concerns about getting SCM to a sufficient level of contraction in a variety of postures, led us to investigate in humans whether cVEMPs could be recorded on one or both of ipsilateral-SCM and contralateral-SPL, relative to the side of the stimulated ear, in a head-turned posture (107) (Figure 6A). This muscle pair acts synergistically for head turns and postures away from the side of the stimulated ear. We found that cVEMPs were recorded significantly more often on contralateral-SPL than ipsilateral-SCM (relative to the side of sound delivery) in a moderately (45°) turned posture, but equally often at more extreme 90° turned postures (Figures 6B,C).

These observations are consistent with a number of points. First, cVEMPs can be expressed on one or both of ipsilateral-SCM or contralateral-SPL, consistent with widespread ramification of vestibulocollic pathways to many neck muscles. Second, the absence of a cVEMP on a particular muscle target does not imply the absence of a vestibulocollic response; instead, the response may simply be evolving on other neck muscles. Such specificity relates to a third point that cVEMPs are best recorded from muscles undergoing tonic recruitment (108); since SCM is only recruited for progressively more extreme turned postures, cVEMPs from SCM are best recorded from such extreme postures. As ipsilateral-SCM but not contralateral-SPL is strongly recruited during the typical clinical posture (subject supine, head elevated, and turned away from the side of stimulation), cVEMPs were expressed robustly on the former but not the latter (Figure 6C). The converse is true (cVEMPs on contralateral-SPL but not ipsilateral-SCM) in a moderately turned head posture. Finally, the cVEMP was expressed as a head-turning response when the subject was in a head-turned posture. Thus, while ACS may initiate a more saccular-dominated response, cervical reflexes initiated by such activation are not constrained to obvious neck flexion/extension synergies, consistent with a flexible mapping of a given vestibular stimulus onto motor output that varies in a position-dependent manner.

Recognizing that the cVEMP can be expressed preferentially on muscles other than SCM may lead to improved assessments of otolith reflexes in specific populations. The clinical posture needed to engender sufficient amounts of SCM recruitment, which as mentioned above involves a supine subject elevating their head and turning it sharply, can be arduous to attain and maintain in very young, old, or infirm populations. Accordingly, cVEMPs from SCM are reliable in healthy adults up to ~60 years old, but far less reliable in a healthy elderly cohort (6). Thus, there is a high proportion of false positive observations in the elderly (where a “positive” observation consists of an absent cVEMP). Recent work of ours (109) has shown that recording cVEMPs from both ipsilateral-SCM and contralateral-SPL in a simple head-turned posture reduces this rate of false positives both in the elderly, and in a cohort of patients with Parkinson's disease (PD). Previously, PD was associated with a low rate of cVEMPs (110). However, our work suggests that lower rates of cVEMPs in PD may reflect difficulties in attaining and maintain sufficient levels of SCM recruitment, or perhaps other motor-related abnormalities, rather than a pathology of vestibular reflexes *per se*. Assessing cVEMPs in multiple muscle targets in a simple head-turned posture may help differentiate true vestibular pathologies from situations where patients simply could not recruit SCM enough to permit expression of the response. At the current time, the value of recording cVEMPs from muscles other than SCM remains to be more fully explored across the lifespan, and across a variety of clinical conditions.

## Conclusions and future directions

Work in animal models has been essential in the understanding of the functional anatomy and physiology underlying VEMPs. Across multiple levels, such work has revealed a level of complexity that is relevant to interpreting the outcome of VEMPs in the clinical domain. At the level of the sensory apparatus, both ACS and BCV activate a complement of hair cells, which although preferentially distributed to a particular end-organ, should not be viewed as exclusive. Further, there is a high degree of convergence between inputs from both otolith organs, and from otoliths and canals, onto second-order neurons within the vestibular nucleus. Multiple excitatory and inhibitory pathways link the vestibular nuclei to extraocular and neck muscle motoneurons, in addition to interfacing within intrinsic circuits within the spinal cord in the case of the cVEMP. Finally, the way in which reflexive responses are elaborated within the motor apparatus is itself influenced by a number of factors including the proportion of muscle fiber, the intensity of the drive to the motor apparatus, and the level of background recruitment of the muscle being recorded.

The vast majority of animal model work pertaining directly to VEMPs has been conducted in anesthetized preparations, addressing for example the basic mechanisms of hair cell transduction, or the anatomical pathways mediating the shortest input-output pathways of vestibular reflexes. However, vestibular reflexes do not operate in isolation, and broader work investigating the vestibular system across a variety of animal models have revealed fundamental features about vestibular processing, including the convergence of multisensory inputs into the vestibular nucleus, and modulation of vestibular reflexes in the context of overall behavior. In light of this, the monkey model in particular is well positioned to investigate VEMPs in an awake preparation, complementing earlier in awake guinea pigs (111), and capitalizing on the closer homologies in comparative anatomy and the amenability of monkeys to behavioral training. Techniques are established to record from a multitude of neck (85) or extraocular (112) muscles with either chronically- or acutely-implanted EMG electrodes, enabling characterization of the VEMPs across multiple targets. Development of an awake and behaving animal model for VEMPs will present some challenges, such as how to encourage a sufficient amount of recruitment. Fortunately, for the cVEMP, even head-restrained monkeys exhibit a pattern of coupling between eye-in-head position and neck muscle recruitment (113) that resembles that seen in humans (114). Monkeys can also be trained to maintain eccentric eye-in-head positions, and are amenable to neck muscle vibration or passive body-under-head rotations (115–118), which could enable investigations of the multimodal interaction between cVEMPs and neck muscle proprioception.

Work in animal models has clearly contributed to the development and interpretation of VEMPs as a means to test vestibular function in clinical populations. Continued work in animal models will refine our understanding of this response at afferent, central, and efferent levels, as they permit a level of systematic control and precision that exceeds that available in humans. The use of multiple animal models from across the animal kingdom will only help to enrich this understanding, and potentially identify new ways of assessing vestibular health [see also (119)]. Such work will be particularly relevant given the move toward assessing vestibular function in the context of neurodegenerative disorders like Parkinson's as noted above, or in other central neurological disorders more widely (120). Proper interpretation of any results coming from such populations hinges on an understanding of how the pathophysiology may affect the expression of vestibular reflexes, and for this it may be useful to assess VEMPs in animal models of such diseases. Animal models would also seem poised to contribute to the assessment of vestibular reflexes for the current generation of cochlear prosthetics, and next-generation vestibular prosthetics, both to assess end organ or afferent nerve sensitivity to stimulation, and to program such devices to limit undesired cross-talk. Fortunately, both feline and primate models of these prosthetics exist (121–123).

## Author contributions

BC and AC conceived of this review. BC wrote the original draft, and BC and AC revised the manuscript.

### Conflict of interest statement

The authors declare that the research was conducted in the absence of any commercial or financial relationships that could be construed as a potential conflict of interest.

## Abbreviations

ACS: air-conducted sound
BC: biventer cervicis
BCV: bone-conducted vibration
COM: complexus
cVEMPs: cervical vestibular evoked myogenic potentials
EMG: electromyography
IA: irregular-firing afferent
LC: longus capitis
LVST: lateral vestibulospinal tract
MVST: medial vestibulospinal tract
OCI: obliquus capitis inferior
OCS: obliquus capitis superior
oVEMPs: ocular vestibular evoked myogenic potentials
RA: regular-firing afferent
RCP maj: rectus capitis posterior major
RCP med: rectus capitis posterior medius
RCP min: rectus capitis posterior minor
SCM: sternocleidomastoid
SPL: splenius capitis
SSC: semispinalis capitis
TRAP: trapezius
VCR: vestibulo-collic reflex
VEMPs: vestibular evoked myogenic potentials
VOR: vestibulo-ocular reflex.
